# Complex Management of a Migrating Inhaled Metallic Foreign Body in a Child: A Multidisciplinary Success

**DOI:** 10.7759/cureus.70023

**Published:** 2024-09-23

**Authors:** Najat Id El Haj, Maha Adnane, Jihane Ziati, Chafik El Kettani, Amal Haoudar

**Affiliations:** 1 Thoracic Surgery, Université Hassan II, Casablanca, MAR; 2 Anesthesia and Critical Care, Cheikh Khalifa International University Hospital, Casablanca, MAR; 3 Anesthesia and Critical Care, Cheikh Khalifa International University Hospital, Mohammed VI University of Health Sciences, Casablanca, MAR

**Keywords:** bronchoscopy, esophagoscopy, inhaled, metallic foreign body, migrating

## Abstract

Foreign body (FB) inhalation in children is a common and potentially life-threatening occurrence encountered in pediatric emergency medicine. A wide range of clinical presentations including often delayed onset of symptoms make it challenging to identify and provide a timely diagnosis. This increases the risk of complications and leads to suboptimal outcomes. For instance, inhalation of sharp objects may lead to perforations and migrations to surrounding structures making it difficult to retrieve the FB as seen in this case. Additionally, the onset of symptoms can vary, making it difficult to diagnose based on a patient's history alone. An unusual case of an inhaled sharp metallic object (dental bur) in a 13-year-old boy that migrated from the left lower thorax to the right perihilar and finally to the gastric lumen is presented a week after the incident. A 13-year-old boy presented to the emergency department with mild symptoms. He was stable with normal chest findings. Previous rigid bronchoscopy failed to localize and remove the FB and the thoracotomy. A post-operative X-ray was done, and the migration of the FB to the right middle lobe was revealed. A flexible bronchoscopy was then performed, again with no positive results. It was finally the abdominopelvic CT scan followed by the gastroduodenal esophagoscopy that allowed us to visualize and remove the FB from the gastric lumen. In this case, we review the literature to emphasize the diagnostic challenges of FB inhalation in children, focusing on key diagnostic clues that assist clinicians in managing this condition.

## Introduction

Foreign body (FB) inhalation in children is a common and potentially life-threatening occurrence encountered in pediatric emergency medicine. Children are at a heightened risk of inhaling foreign objects, leading to respiratory obstruction and subsequent complications.

The epidemiology of FB inhalation in children reveals a notable prevalence, with studies indicating a higher incidence among toddlers and preschool-aged children, typically between one and three years old [1]. According to Chen et al., the infant mortality rate from FBA has decreased significantly, despite FB inhalation remaining one of the main causes of infantile deaths [2].

Sharp FBs (e.g., bones, safety pins, and needles) can cause perforation and migrate to surrounding structures, causing further injuries.

Clinical presentation varies depending on the nature of the FB, its location within the respiratory tract, and the duration since inhalation. Saki et al. showed that common signs and symptoms include sudden onset of coughing, choking, wheezing, stridor, dyspnea, or respiratory distress [3]. However, in some cases, the presentation may be subtle, with nonspecific symptoms such as persistent cough, recurrent respiratory infections, or unexplained fever, making diagnosis challenging.

Managing a migrating FB inhalation in children presents significant challenges due to the difficulty in localizing the object, which can move unpredictably within the respiratory tract, complicating diagnosis. The ultimate challenge lies in pinpointing its exact location through imaging and endoscopy. Removal procedures are fraught with risks, including the potential for airway damage, obstruction, or accidental displacement of the FB into more sensitive areas. These complications can lead to severe respiratory distress, infections, or long-term pulmonary issues if not promptly and effectively addressed.

## Case presentation

A 13-year-old boy without any pertinent medical history initially presented to the emergency department a week after inhaling a metallic FB, presenting mild symptoms: recurrent cough and nausea. The incident occurred during a dental appointment when the dental bur fell and was accidentally inhaled by the patient.

**Figure 1:**
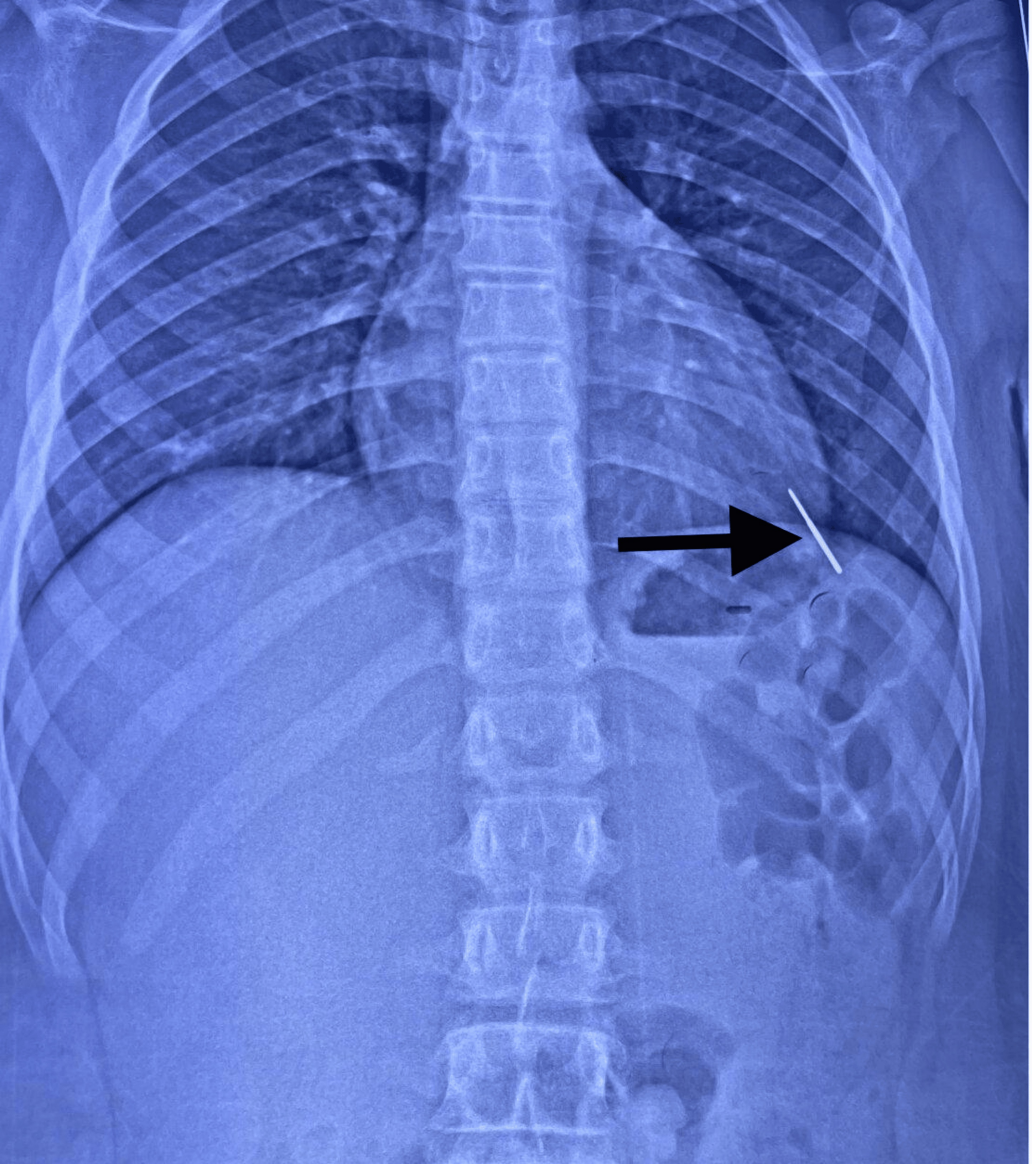
Chest X-ray showing a FB in the left base of the thorax (06/05/2024) FB, foreign body

He was initially taken to a local hospital in his hometown, where a chest X-ray revealed an FB in the left lower thorax with no evidence of air or fluid pleural effusion (Figure 1).

**Figure 2:**
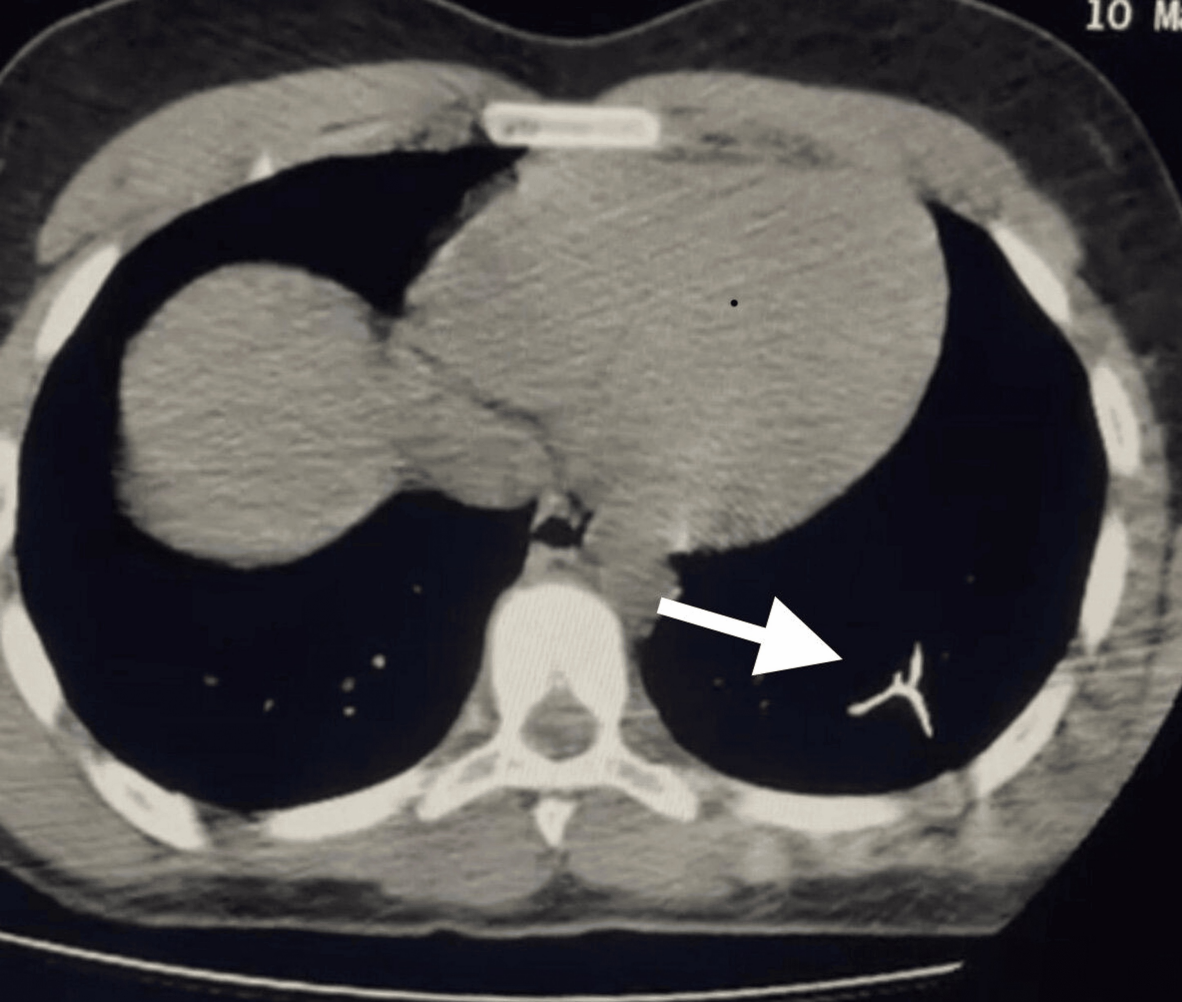
Chest CT scan showing a FB in the left basal lung (10/05/2024) FB, foreign body

A gastroduodenal esophagoscopy was performed, but no FB was visualized or removed. A subsequent thoracic CT scan showed that the dental bur was in the inferior lung lobe (Figure 2).

One week later, the patient was referred to our hospital for further management. Laboratory investigation (Table 1) and rigid bronchoscopy were performed, but the procedure failed to visualize the FB. The patient was then admitted to the pediatric intensive care unit, intubated and ventilated due to complications during the bronchoscopy, where he experienced severe bronchospasm and alveolar hemorrhage.

**Table 1:** Laboratory investigation report (main assessments)

	16/05/2024	19/09/2024
White cell count (per μL)	22020	14000
Neutrophil count (per μL)	18390	7300
Lymphocyte count (per μL)	1300	1250
Platelet count (per μL)	403000	380000
Hemoglobin (g/dL)	12.7	13.1
Hematocrit (%)	39.6	40
CRP (mg/L)	12.40	6.1
PCT (ng/mL)	1.90	0.2
Sodium (mEq/L)	140	139
Potassium (mEq/L)	3.5	3.4
Alkaline reserve (mEq/L)	22	22
Prothrombin ratio (%)	95.9	96
INR	1.02	1.03

**Figure 3:**
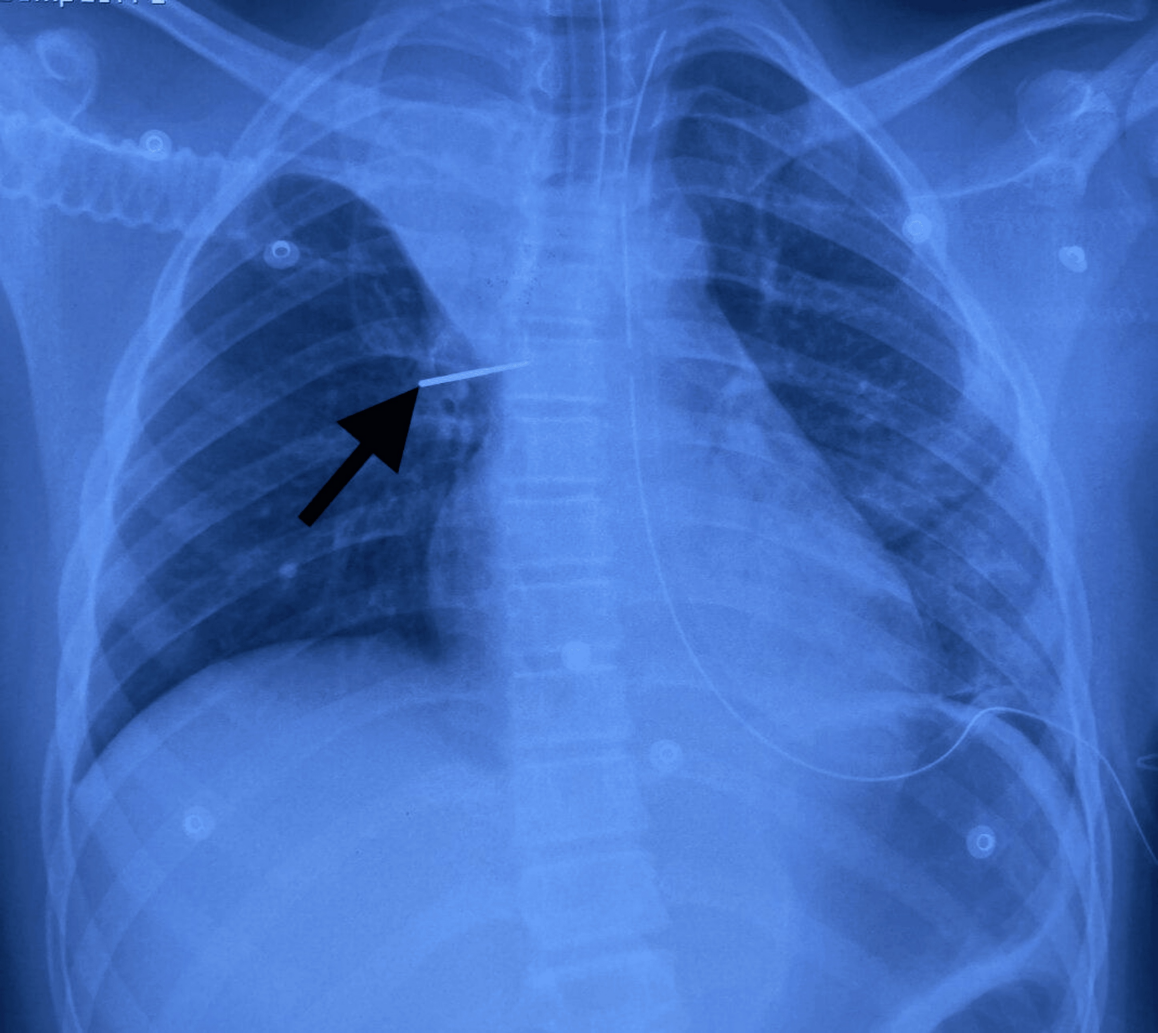
Chest X-ray showing a right perihilar FB (16/05/2024) FB, foreign body

The patient was extubated the day after his admission. A second chest X-ray (Figure 3) showed the migration of the FB, which was still in the inferior left lobe; thoracic surgeons were involved in surgical removal. Initially, video-assisted thoracoscopic surgery (VATS) was planned, but when the FB could not be located, the procedure was converted intraoperatively to a thoracotomy. All segments were palpated, but the FB was not found. 

**Figure 4:**
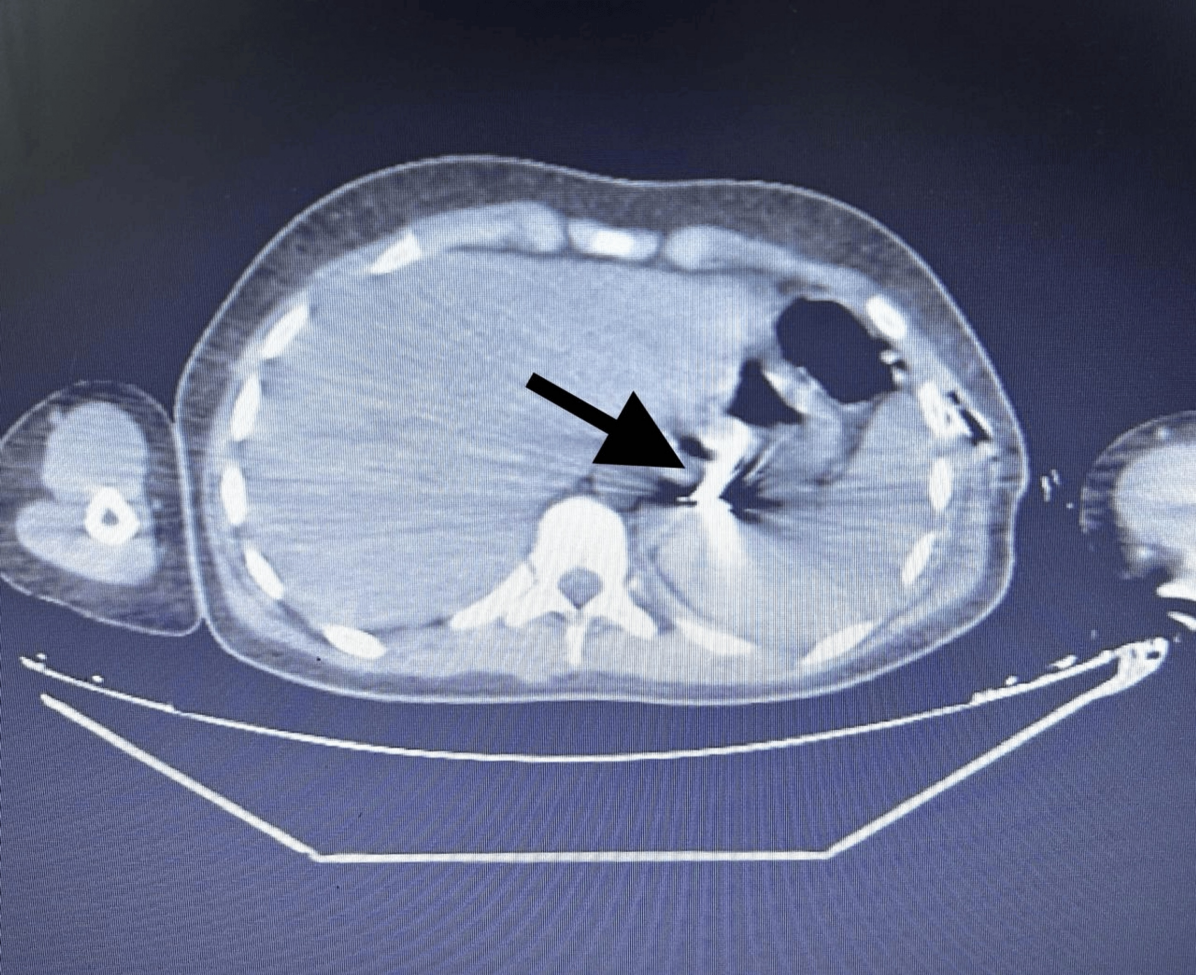
Abdominopelvic CT scan showing a FB in the gastric lumen

**Figure 5:**
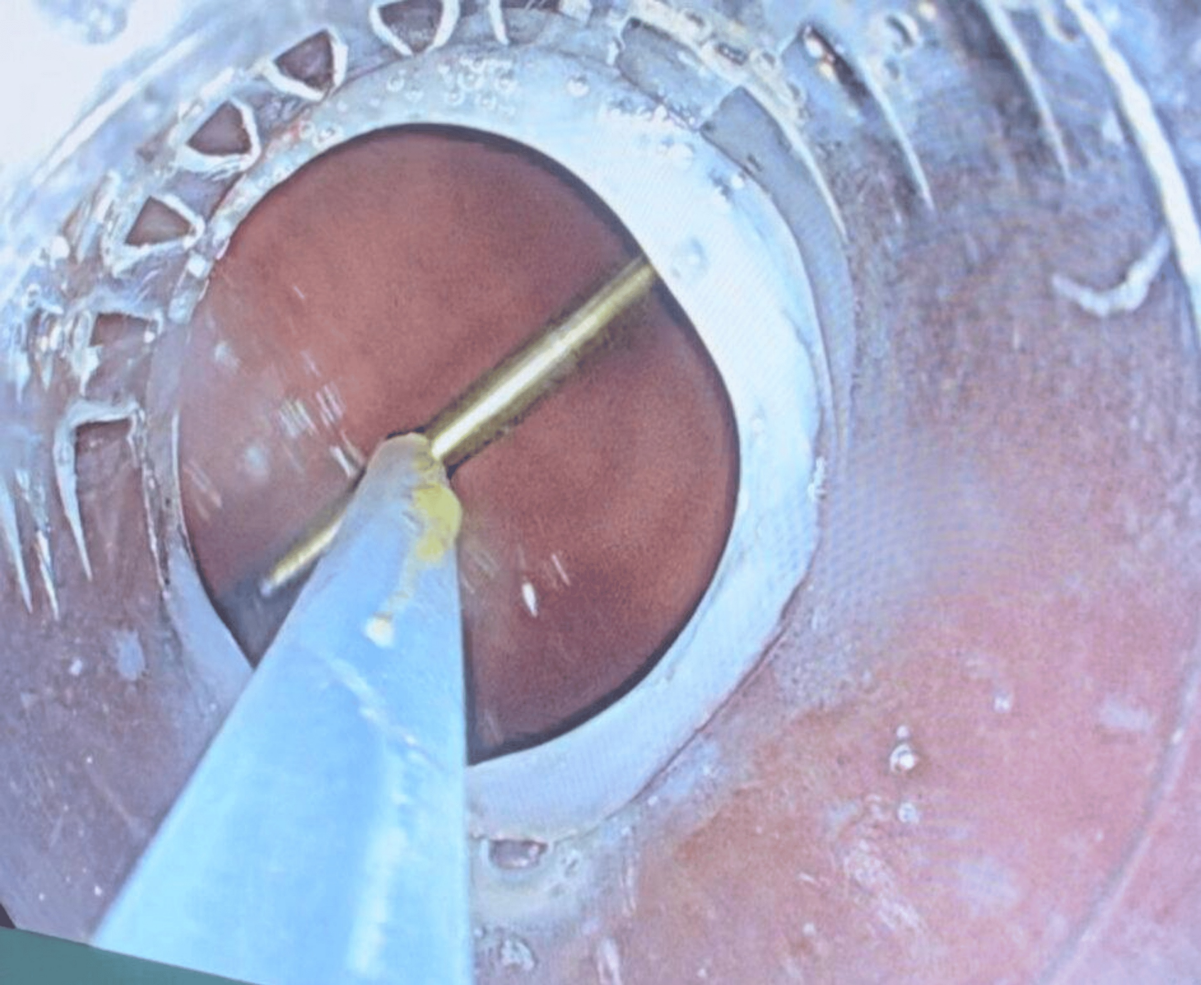
Removal of the metallic FB from the gastric lumen during the gastroduodenal esophagoscopy procedure

A third chest X-ray was done and visualized a migration of the FB to the right middle lobe. A flexible bronchoscopy was performed for one hour following the X-ray, again with no positive result. Immediately afterward, an abdominopelvic CT scan (Figure 4) revealed that the FB had migrated to the gastric lumen. The gastroenterology team was then requested to perform a gastroduodenal esophagoscopy, which successfully removed the FB (Figure 5).

The child was discharged from the hospital the day after the removal.

## Discussion

Foreign body inhalation represents a significant challenge in emergency pediatric care, with the potential for serious morbidity and mortality especially when rapid migration occurs [4].

In this case of a 13-year-old boy who inhaled a metallic dental bur, we will discuss the complexities involved in diagnosing and locating foreign bodies that migrate post-inhalation, as well as the therapeutic challenges encountered in its management. The shape and chemical nature of the FB are two important factors in evaluating the risk of migration [5]. Most inorganic foreign bodies, such as the dental drill in this case, do not result in any prominent symptoms [6].

Diagnostic challenges

The diagnosis of inhaled FBs can be particularly challenging. While chest radiography remains the initial diagnostic modality of choice, its sensitivity is limited, particularly for non-radiopaque objects or those that have migrated. The study by Kripa et al. stated that for the chest X-rays performed in all 98 patients, 54.1% of patients had normal chest X-rays, radiopaque FB was seen for 24% of the patients, atlectasis in 11%, and consolidation in 7% [7]. This is similar to the studies done by Eren et al. who also found that two-thirds of their patients had normal chest X-rays [8].

In this case, the initial chest X-ray identified the FB in the left lower lung lobe, yet subsequent procedures failed to visualize it. This highlights the need for a high index of suspicion and the use of advanced imaging modalities when initial diagnostic efforts are inconclusive. CT is recommended as it offers superior resolution and can help in accurately localizing FBs, especially when they migrate within the thoracic cavity or beyond. Studies indicate that CT has a higher diagnostic yield compared to standard radiography in cases of suspected FB aspiration.

Utilization of bronchoscopy

Bronchoscopy remains the gold standard for diagnosing and managing migrating foreign bodies. It allows direct visualization of the airways, precise localization of the FB, and immediate intervention if extraction is warranted. Early bronchoscopic examination is crucial in cases where clinical suspicion remains high despite inconclusive imaging results. However, the nature of some FB makes them migrate easily, which complicates the extraction by endoscopy and thereby leads to thoracotomy [9]. Failure of rigid bronchoscopy combined with flexible bronchoscopy can occur, which is the case here [9].

Complications of bronchoscopy

Bronchoscopy is the gold standard for both diagnosis and removal of inhaled FBs. However, it is not without risks, particularly in pediatric patients who are more susceptible to complications such as bronchospasm, hypoxemia, and airway trauma. Kaur et al. have reported hypoxia complication rate to be 10% of bronchoscopy’s complications [10]. In this case, the patient experienced a severe bronchospasm during the bronchoscopy procedure, necessitating intubation and mechanical ventilation in the intensive care unit. The presence of a skilled multidisciplinary team, including anesthesiologists and pediatric intensivists, is crucial to managing such complications effectively.

Multidisciplinary approach and surgical intervention

The management of this case necessitated the involvement of multiple specialties. Initial rigid bronchoscopy failed to visualize or retrieve the FB, leading to the involvement of thoracic surgeons. The decision to convert from video-assisted thoracoscopic surgery (VATS) to an open thoracotomy reflects the complexity and unpredictability of FB management. Although VATS is less invasive and generally preferred, it may not always be feasible, particularly when the FB is not readily visible or accessible. The conversion to open surgery, while more invasive, allows for thorough exploration and palpation of all thoracic segments, a critical step when other methods fail. To avoid unnecessary pulmonary resections, it is mandatory to confirm the precise location of an intrapulmonary FB; the aforementioned especially holds true in the case of inorganic and simple-shaped airway foreign bodies because of the possibility of an unexpected migration. The presumption that a bronchoscopically irremovable airway FB is firmly fixed in the bronchial tree and does not migrate intraoperatively is incorrect.

Migration of the foreign body

Although rare, migration of FBs has been documented in the literature [11]. The spontaneous movement of the FB underscores the dynamic nature of the pediatric respiratory and gastrointestinal systems. This phenomenon necessitates a comprehensive and adaptable diagnostic approach. The follow-up abdominopelvic CT was instrumental in identifying the new location of the FB, highlighting the importance of repeated and varied imaging studies in complex cases.

Endoscopic retrieval

Endoscopic retrieval of gastrointestinal FBs is well-documented as an effective and minimally invasive approach. The eventual successful removal of the FB via gastroduodenal esophageal fibroscopy aligns with best practices. Endoscopic techniques are preferred for their high success rates and lower risk profiles compared to surgical interventions. The literature supports the use of flexible endoscopy for FB retrieval in the gastrointestinal tract due to its ability to reach and remove objects with minimal patient discomfort and rapid recovery times [12].

## Conclusions

This case underscores the complexity and multidisciplinary nature of managing inhaled foreign bodies in pediatric patients. It illustrates the major diagnostic challenge to precisely determine the localization of the FB due to its unpredictable migration (from the chest to the gastric lumen in this case). Therefore, just like in other cases and studies, the patient had to undergo several imaging and intervention techniques such as repeated bronchoscopy (rigid and flexible), thoracotomy, and gastroduodenal esophagoscopy to finally visualize and remove the FB. This leads us to question the indications at each intervention as well as the patient risk-benefit balance. The case reported here shows that in order to avoid unnecessary interventions, it is crucial to confirm the precise location of the FB and always consider possible migration in case of failed visualization of the FB.
